# Large Language Models Accurately Identify People Who Inject Drugs From Infectious Diseases Discharge Summaries in an Australian Hospital

**DOI:** 10.1111/dar.70169

**Published:** 2026-05-14

**Authors:** David Goodman‐Meza, Marianne Martinello, Jeffrey Masters, Danielle Russell, Brendan Jacka, Gail V. Matthews, Gregory J. Dore

**Affiliations:** ^1^ St Vincent's Hospital Sydney Australia; ^2^ Kirby Institute UNSW Sydney Sydney Australia; ^3^ Prince of Wales Hospital Sydney Australia; ^4^ Royal Prince Alfred Hospital Sydney Australia

## Abstract

**Introduction:**

People who inject drugs (PWID) face a high risk for serious infections, yet International Classification of Diseases (ICD) codes fail to identify this population. Large language models (LLM) offer a promising alternative by extracting information from unstructured clinical text. This study evaluated the diagnostic performance of off‐the‐shelf LLMs in identifying PWID and related attributes from hospital discharge summaries.

**Methods:**

In this cross‐sectional study, discharge summaries from the Infectious Diseases service at St Vincent's Public Hospital, Sydney, between 2018 and 2022 were reviewed. A single reviewer manually annotated each de‐identified summary for PWID status, drugs reported, injection recency and opioid agonist therapy. Eight LLMs (Gemma3, Llama 3.3, Mistral, Phi4, hippomistral, llama3‐med [8B and 70B] and OpenBioLLM) were compared using prevalence‐weighted average‐F1 scores. Diagnostic metrics with bootstrapped 95% confidence intervals were calculated for each annotated category.

**Results:**

Of 859 first admissions, manual review identified 149 (17.1%) PWID. ICD codes showed low sensitivity (≤ 0.32) but high specificity (≥ 0.97) for identifying PWID. The best‐performing model (Llama 3.3) achieved a prevalence‐weighted average‐F1 of 0.845 (0.733, 0.927). For injecting drug use, sensitivity was 0.819 (95% CI 0.753, 0.879) and specificity 0.999 (0.996, 1.00). Identification of heroin, methamphetamine, cannabis and methadone was near perfect (F1 > 0.973), while illicit prescription opioid and benzodiazepine use were identified less accurately (F1 = 0.400 and 0.606).

**Discussion and Conclusions:**

LLMs accurately identify PWID from discharge summaries, outperforming ICD codes. Challenges remain for certain substances, underscoring the need for task‐specific tuning, external validation and integration with structured data to enhance surveillance and interventions.

## Key Points

- Large language models accurately identified people who inject drugs (PWID) from hospital discharge summaries, outperforming International Classification of Diseases codes.
- The top‐performing off‐the‐shelf model (Llama 3.3) achieved high sensitivity (0.82) and near‐perfect specificity (0.999) for PWID identification.
- Identification of commonly used substances (heroin, methamphetamine, cannabis, methadone) was accurate, whereas illicit prescription opioid and benzodiazepine use remained challenging.
- International Classification of Diseases codes missed the majority of PWID cases (F1 ≤ 0.43).
- Further work is needed to fine‐tune models, validate externally and integrate structured data to enhance clinical and public health utility.

## 1 Introduction

People who inject drugs (PWID) face an increased risk of developing bacterial infections that lead to hospitalisations, notably skin and soft tissue infections, infective endocarditis, and bone and joint infections [1, 2]. These infections can lead to significant morbidity, mortality and healthcare costs [3]. In Australia, injecting drug use remains common, with national estimates suggesting 1.4% of people over 14 years of age have injected drugs in their lifetime [4], and ongoing increases in injection‐related hospital admissions reported over the past decade [5, 6]. These admissions represent critical opportunities for surveillance, intervention and linkage to harm‐reduction and addiction treatment services. Accurate identification of PWID within hospital electronic health records (EHR) is therefore essential for epidemiologic monitoring, clinical decision‐making and evaluation of public health responses.

Despite this need, identifying PWID using routinely collected EHR data remains methodologically challenging. Traditional methods of identifying PWID within hospital EHRs have relied on International Classification of Diseases (ICD) codes [5, 6, 7, 8, 9]. However, these codes often lack specificity and may not accurately capture this patient population [8, 9, 10, 11]. Previous work demonstrated that natural language processing (NLP) and supervised machine learning techniques applied to clinical notes identify PWID with greater accuracy than ICD codes alone [11]. However, this work was primarily focused on identification of PWID without considering other characteristics such as the specific drugs used, temporality of injection or use, or whether treatment was provided. This research was conducted within the U.S. Veterans Administration hospitals, leaving its diagnostic performance in other settings, including Australian hospitals, unclear.

There has been a rapid evolution in NLP with the introduction of large language models (LLM) [12]. LLMs are advanced artificial intelligence systems designed to comprehend and generate human language by processing vast amounts of text data. These models, such as OpenAI's GPT series [13] and Meta's LLAMA [14], utilise deep learning techniques to perform a wide range of NLP tasks, including text generation, translation and summarisation. Unlike traditional NLP methods that often rely on task‐specific algorithms such as classifying one entity and require large amounts of data to train models, LLMs offer a more generalised approach, enabling them to adapt to various language tasks with minimal to no fine‐tuning [12, 13, 14, 15]. LLMs can leverage contextual inference to interpret complex clinical language, including indirect references, abbreviations, negation and temporal qualifiers [12, 13]. This capability is particularly relevant for identifying PWID, where documentation is often fragmented across clinical sections, varies substantially between clinicians and requires distinguishing illicit from prescribed drug use and current from historical injection.

The performance of off‐the‐shelf LLMs for identifying PWID and related clinical characteristics in Australian hospital EHRs has not been systematically evaluated. In particular, it remains unclear whether LLMs can accurately identify not only injecting drug use itself, but also specific substances used, recency of injection and receipt of opioid agonist therapy, or how their performance compares with ICD‐based approaches. In this study, we evaluated the diagnostic performance of multiple locally deployed, open‐source LLMs in identifying PWID from infectious diseases discharge summaries at a tertiary Australian hospital. Our objectives were to [1] compare LLM‐based identification of PWID with ICD‐10 code–based methods, [2] assess LLM performance in extracting drug‐specific and treatment‐related characteristics and [3] examine model performance across key demographic subgroups to assess fairness. By addressing these gaps, this work aimed to clarify the utility and limitations of LLMs for clinical surveillance and research in substance use.

## 2 Methods

### 2.1 Data Source

This was a cross‐sectional study conducted at St Vincent's Hospital (SVH), Sydney, Australia, a tertiary inner‐city public teaching hospital. We included cases that were discharged from the Infectious Diseases service between 1 January 2018 and 31 December 2022. The Infectious Diseases service manages complex bacterial, viral and fungal infections, including injection‐related infections such as skin and soft tissue infections and infective endocarditis, making it a clinically relevant setting for evaluating methods to identify PWID. Only the first hospitalisation to the service was considered. Discharge summaries (notes) were extracted from SVH's EHR in portable document format (PDF). All notes were stripped of identifiers, institutional boilerplate text, document artefacts (e.g., hospital headers and footers, page numbers, printing timestamps and corporate identifiers). De‐identification was done programmatically using a custom rule‐based preprocessing pipeline. Direct identifiers and quasi‐identifiers were then systematically replaced with standardised placeholder tokens to preserve sentence structure while removing identifying information. Specifically, patient names were replaced with [NAME]; dates (including admission dates, discharge dates and dates of birth) with [DATE]; medical record numbers with [MRN]; individual healthcare identifiers with [IHI]; telephone numbers with [PHONE]; and postal addresses with [ADDRESS]. The remaining text was normalised by collapsing excess whitespace. No further preprocessing was done (e.g., removal of numbers, unifying the text case, correction of misspellings). Notes were imported into a local instance of Label Studio 1.13 (HumanSignal, San Francisco, USA) for annotation (see Gold Standard below). Metadata related to the admission were received from the SVH Medical Records Department and included age, sex, Aboriginal or Torres Strait Islander status, length of stay, discharge status and ICD codes for the admission. The report was written in line with the TRIPOD‐LLM reporting guideline [16]. The study was approved by the SVH Human Research Ethics Committee with a waiver of informed consent.

### 2.2 Gold Standard

The gold standard served as the ground truth against which LLM and ICD‐based classifications were evaluated and was determined by manual review. All notes were reviewed by a single author (DGM). The author annotated each note based on a prespecified annotator guide (Supplemental Table 1) to identify language related to if the individual was a PWID, the drug they reported using (e.g., heroin, methamphetamine, cocaine, prescription opioids, benzodiazepines, cannabis), if injecting drug use was current (within last 30 days) or historical, and whether opioid agonist therapy was prescribed (methadone or buprenorphine).

### 2.3 International Classification of Diseases Codes

All primary and secondary ICD‐10 codes documented in the EHR were considered for each admission. As no specific ICD‐10 code exists to identify PWID, combinations of code groups were evaluated as a proxy to identify injecting drug use based on prior literature [7, 11] and modified to the Australian ICD‐10 version (ICD‐10‐AM). Code groups included: HIV (B21 to B24, Z21), hepatitis C (B17.1, B18.2, Z22.52), substance use‐related (Z72.2, Z86.41, T40.1 to T40.5, T43.6), substance‐use disorders (F11, F14, F15, F19) and homelessness (Z59). Specific substances were also attempted to be identified by ICD codes: Heroin (T40.1, F11), prescription opioids (T40.2 to T40.4, F11), methamphetamine (T43.6, F15) and cocaine (T40.5, F14).

### 2.4 Classification Task

The modelling task was formulated as a multi‐label binary classification problem, in which each discharge summary was independently evaluated for the presence or absence of multiple clinically relevant attributes. For each note, models were prompted to return a set of binary (true/false) outputs corresponding to predefined labels. These labels comprised four groups of related classification tasks: (i) injecting drug use, defined as any documentation of current or historical injecting drug use (binary: yes/no); (ii) injection chronicity, evaluated using two non‐mutually exclusive binary labels indicating current injection (within 30 days) and historical injection (remote use); (iii) substance‐specific use, evaluated as independent binary labels for each substance (e.g., heroin, methamphetamine, prescription opioids, benzodiazepines, cannabis), allowing for concurrent use of multiple substances; and (iv) treatment for opioid use disorder, evaluated using binary indicators for methadone and buprenorphine prescription.

### 2.5 Selection of LLMs

Open‐source NLP models were selected that could be run on local computers to protect highly sensitive data. No data was submitted to commercial servers. All analyses were performed within the R programming environment using the rollama package [17], which interfaces with Ollama (Ollama Inc., Palo Alto, US), a local deployment framework for running LLMs on personal hardware [18]. This framework enabled secure, offline inference with open‐source LLMs facilitating reproducible and privacy‐preserving NLP classification workflows for clinical text. LLMs were selected based on public availability for local deployment, feasibility of offline inference and prior evidence of performance in general and biomedical NLP tasks using Elo ratings. Elo ratings [19, 20] are a dynamic ranking metric that reflects a model's relative performance through head‐to‐head comparisons across standardised tasks. In the NLP context, higher Elo scores indicate superior performance against other models on benchmark challenges. Elo ratings were used as a secondary, pragmatic benchmark to guide selection among candidate models, but were not assumed to reflect task‐specific performance for EHR‐based substance use identification. General LLMs included *Gemma3* (Google), *Llama 3.3* (Meta), *phi4* (Microsoft) and *Mistral* (Mistral AI). Pre‐fine‐tuned medical LLMs included *hippomistral*, *llama3‐med* and *OpenBioLLM‐Llama3* (see Supplemental Table 2 for model details including parameter size and families). All evaluations were performed with LLMs without any additional fine‐tuning to this task. A prompt was developed describing the classification task for each condition to be identified by the models. Prompt development was informed by prior work conducted in the United States on LLM‐based identification of substance use in clinical notes [15] and was refined to align with the prespecified annotator guide and the Australian clinical context (see Supplemental Table 3 for full prompt).

### 2.6 Error Analysis

A detailed error analysis was performed on the outputs of the top‐performing LLM to characterise the types and sources of misclassification. All false positives and false negatives were manually examined and the reason for the error categorised. Categories were determined post hoc during review. Error categories included: missed documentation, where relevant information was present but not identified by the model; temporal misclassification, where current and historical injecting drug use were incorrectly distinguished; contextual misinterpretation, including confusion between illicit and prescribed medication use, misattribution of third‐party substance use, or interpretation of laboratory findings without explicit clinical context; and human annotation error, identified upon re‐review of the gold‐standard label. The error analysis was conducted by a clinician–researcher (DGM) with expertise in infectious diseases, substance use disorders and clinical documentation, who also performed the initial gold‐standard annotation. Error counts were summarised descriptively by reason for the error.

### 2.7 Statistical Analysis

Descriptive statistics were used to summarise and compare individuals identified as PWID with those not identified as PWID, with continuous variables reported as medians and interquartile ranges, and categorical variables as counts and percentages, using *t*‐tests, Mann–Whitney U tests, or Fisher's exact tests, as appropriate. As no training of models was done, all data was used as a test set. Diagnostic performance metrics, including sensitivity, specificity, positive predictive and negative predictive values, and F1 score, were calculated for both LLMs and ICD code‐based identification methods. For ICD‐10 code–based approaches, performance was evaluated in identifying PWID (via proxy combinations) and in detecting heroin, prescription opioids, methamphetamine and cocaine. Temporality (current or historical) was not evaluated for ICD‐10 codes. For LLM‐based approaches, performance was evaluated in identifying PWID, in detecting heroin, prescription opioids, methamphetamine and cocaine, acuity of use, and treatments for substance use disorder (methadone and buprenorphine).

Given that LLMs were tasked with identifying multiple binary labels within the same discharge summary, the prevalence‐weighted average F1 score was used as the primary criterion to evaluate and select the best‐performing model, with label‐specific diagnostic metrics examined subsequently to inform clinical interpretation. The F1 score balances a model's sensitivity (recall) and positive predictive value (precision) for each condition. Average (or macro) F1 score calculates the unweighted mean of per‐class F1 scores, treating all classes equally. In contrast, prevalence‐weighted average F1 score accounts for class imbalance by weighting each class' F1 score by its prevalence, ensuring that more prevalent conditions have a proportionate impact on the overall score. In a multi‐label setting where models are required to identify numerous attributes from the same clinical note, this approach provides a pragmatic summary of overall performance across tasks with markedly different base rates. Importantly, the intent of prevalence weighting is not to down‐weight clinically important but rare outcomes, but rather to avoid an aggregate metric being dominated by unstable estimates from extremely low‐prevalence labels. Low‐prevalence labels were retained in the aggregate metric to preserve completeness of the multi‐label evaluation, although should be interpreted cautiously. Instability related to small sample sizes was addressed through reporting of bootstrapped 95% confidence intervals for all performance metrics. Ninety‐five percent confidence intervals (CI) for all performance metrics were derived using bootstrapping techniques with 1000 iterations. All analyses were performed in R 4.3.3 (R Foundation for Statistical Computing, Vienna, Austria).

### 2.8 Fairness Analysis

To evaluate potential algorithmic bias across demographic subgroups, a fairness analysis was conducted of the best‐performing NLP classifier for identifying injecting drug use [21]. Fairness metrics were calculated for key subgroups defined by age (dichotomised at the median), sex (male vs. female) and Aboriginal and/or Torres Strait Islander identification. We operationalised three broad group fairness criteria: independence (demographic parity, equal predicted positive rates), separation (equality of opportunity via true positive rate parity and equalised odds via both true positive and true negative rate parity) and sufficiency (predictive parity via predictive values) [22]. In addition, the Matthews correlation coefficient was reported to capture balanced model performance across groups (a description of metrics and formulas to calculate each are available in Supplemental Table 4). For each subgroup, parity ratios (minimum to maximum value across groups) were calculated, with ratios between 0.80 and 1.25 considered consistent with acceptable fairness under the “80% rule” [23].

## 3 Results

Between 2018 and 2022, there were 998 individual hospitalisations considered for inclusion. Of these, 141 were readmissions and excluded. The final analysis dataset included 859 individual patients and their first hospitalisation during the study period. The mean number of words per discharge summary was 300 with a standard deviation of 182 words. Notes pertaining to PWID were significantly longer (mean words 350 vs. 290, *p* < 0.001).

Table 1 shows demographic, hospitalisation and drug use‐related characteristics by PWID status identified during manual review. Manual review identified 149 (17.1%) as PWID. PWID were younger at admission (45 vs. 54 years, *p* < 0.001), more likely to identify as Aboriginal or Torres Strait Islander (24 vs. 5.6%, *p* < 0.001), had a longer length of stay (6 vs. 4 days, *p* < 0.001) and were more likely to direct their own discharge (23% vs. 4.5%) compared to the non‐PWID group. PWID and non‐PWID had a similar proportion of ID admissions for skin and soft tissue infections, sepsis and pneumonia, but PWID had a higher proportion of admissions due to unspecified bacterial infections and endocarditis than non‐PWID. PWID had a lower proportion of admissions due to unspecified fever, urinary tract infections or unspecified viral infections than non‐PWID. HIV prevalence was low in this sample and did not differ meaningfully between groups, with one case of HIV identified among individuals classified as PWID and one case among those not classified as PWID. Among PWID, methamphetamine was the most common substance reported in the notes (31%), followed by heroin (24%), cannabis (9.5%) and prescription opioids (8.8%). Close to half of PWID were prescribed methadone (40%) or buprenorphine (9.5%) for treatment of opioid use disorder. Of note, only 2 cases had an ICD code indicative of a substance use disorder.

**TABLE 1:** Demographic and clinical characteristics of individuals admitted to the infectious diseases service at St Vincent's Hospital, Sydney, between 2018 and 2022 (*n* = 859).

	Non‐PWID, *n* = 710	PWID, *n* = 149	*p*
Age, median (IQR)	54 (39, 68)	45 (36, 50)	< 0.001
Sex			> 0.9
Male	485 (68%)	101 (68%)	
Female	225 (32%)	48 (32%)	
Aboriginal and/or Torres Strait Islander status			< 0.001
Non‐Indigenous	665 (94%)	114 (77%)	
Aboriginal and/or Torres Strait Islander status	41 (5.8%)	35 (23%)	
Unknown	4 (0.6%)	0 (0%)	
Length of stay, days	4 (2, 8)	6 (3, 13)	< 0.001
Discharge status			< 0.001
Discharge to home	607 (85%)	103 (69%)	
Transfer	68 (9.6%)	13 (8.7%)	
Patient‐directed discharge	33 (4.6%)	33 (22%)	
Death	3 (0.4%)	0 (0%)	
Primary diagnosis			
Skin and soft tissue infections	179 (25%)	41 (28%)	0.6
Fever	40 (5.6%)	1 (0.7%)	0.01
Sepsis	28 (3.9%)	9 (6.0%)	0.3
Urinary tract infections	31 (4.4%)	0 (0%)	0.009
Bacterial infections	12 (1.7%)	9 (6.0%)	0.005
Viral infection	21 (3.0%)	0 (0%)	0.04
Pneumonia	19 (2.7%)	1 (0.7%)	0.2
Endocarditis	7 (1.0%)	10 (6.7%)	< 0.001
Other	373 (53%)	78 (52%)	> 0.9
Substances			
Heroin	0 (0%)	36 (24%)	< 0.001
Prescription opioids (misused)	1 (0.1%)	13 (8.8%)	< 0.001
Fentanyl (misused)	0 (0%)	3 (2.0%)	0.005
Methamphetamine	14 (2.0%)	47 (32%)	< 0.001
Cocaine	0 (0%)	3 (2.0%)	0.005
Cannabis	4 (0.6%)	14 (9.4%)	< 0.001
Opioid agonist therapy			
Methadone	28 (3.9%)	60 (40%)	< 0.001
Buprenorphine	2 (0.3%)	13 (8.7%)	< 0.001

*Note:* Values represent *n* (%) unless noted.

Abbreviations: IQR, interquartile range; PWID, people who inject drugs.

Table 2 presents diagnostic performance for ICD‐10–based identification. Across the composite indicators, overall performance was low (highest F1 score was 0.431) despite high specificity. The definition combining hepatitis C and substance use disorder codes achieved an F1 of 0.431, with sensitivity of 0.315 and positive predictive value of 0.681. Increasing the number of concurrent diagnostic categories (3 or more ICD codes) improved specificity (≥ 0.989) but further reduced sensitivity (< 0.107), resulting in F1 values of 0.185. For substance‐specific codes, performance was again low. Heroin‐related ICD codes demonstrated moderate performance (F1 = 0.339), while prescription‐opioid codes showed weaker results (F1 = 0.129). Methamphetamine‐related ICD codes failed to identify any true cases.

**TABLE 2:** Diagnostic metrics of International Classification of Diseases codes at identifying injecting drug use (*n* = 859).

Category	Predicted	F1 score^a^	Accuracy	Sensitivity	Specificity	PPV	NPV
Injecting drug use (*n* = 149)							
Hepatitis C and substance use disorder	69	0.431 [0.348, 0.511)	0.856 (0.833, 0.880)	0.315 (0.241, 0.394)	0.969 (0.957, 0.982)	0.681 (0.574, 0.794)	0.871 (0.849, 0.894)
HIV and substance use disorder	0	NA	0.827 (0.803, 0.850)	0.000 (0.000, 0.000)	1.000 (1.000, 1.000)	NA	0.827 (0.803, 0.850)
≥ 3 of HIV, hepatitis C, substance use disorder, substance use‐related	1	0.013 (0.012, 0.043)	0.828 (0.803, 0.851)	0.007 (0.000, 0.021)	1.000 (1.000, 1.000)	1.000 (1.000, 1.000)	0.828 (0.803, 0.851)
≥ 3 of HIV, hepatitis C, substance use disorder, substance use‐related or homelessness	24	0.185 (0.106, 0.263)	0.836 (0.813, 0.861)	0.107 (0.058, 0.160)	0.989 (0.980, 0.996)	0.667 (0.467, 0.852)	0.841 (0.817, 0.866)
≥ 4 of HIV, hepatitis C, substance use disorder, substance use‐related or homelessness	1	0.013 (0.012, 0.048)	0.828 (0.800, 0.855)	0.007 (0.000, 0.022)	1.000 (1.000, 1.000)	1.000 (1.000, 1.000)	0.828 (0.800, 0.854)
Heroin (*n* = 36)	128	0.339 (0.245, 0.426)	0.873 (0.852, 0.895)	0.757 (0.610, 0.875)	0.878 (0.856, 0.900)	0.219 (0.150, 0.289)	0.988 (0.979, 0.995)
Prescription opioids (*n* = 14)	129	0.140 (0.066, 0.221)	0.857 (0.834, 0.881)	0.714 (0.461, 0.933)	0.859 (0.836, 0.883)	0.078 (0.035, 0.128)	0.995 (0.989, 0.999)
Methamphetamine (*n* = 61)	0	NA	0.929 (0.913, 0.945)	0.000 (0.000, 0.000)	1.000 (1.000, 1.000)	NA	0.929 (0.913, 0.945)

*Note:* Numbers denote point estimates with 95% bootstrap confidence intervals (1000 iterations). Cocaine and fentanyl were evaluated as separate substance categories but are not reported due to low numbers (*n* = 3 for each).

Abbreviations: NPV, negative predictive value; PPV, positive predictive value.

Codes: HIV (B21 to B24, Z21), hepatitis C (B17.1, B18.2, Z22.52), substance use‐related (Z72.2, Z86.41, T40.1 to T40.5, T43.6), substance‐use disorders (F11, F14, F15, F19) and homelessness (Z59). Heroin (T40.1, F11), prescription opioids (T40.2 to T40.4, F11) and methamphetamine (T43.6, F15).

^a^ F1 score is a summary measure of diagnostic performance that balances sensitivity and positive predictive value, reflecting a model's ability to correctly identify true cases while minimising false positives.

Table 3 summaries and compares the diagnostic performance of different LLMs by prevalence‐weighted average F1 scores derived from 1000 bootstrap samples. The best performing model was llama3.3 with a prevalence‐weighted average F1 of 0.845, followed by llama3‐med42‐70b (F1 = 0.808), OpenBioLLM‐Llama3‐70B (F1 = 0.792), mistral (F1 = 0.772), llama3‐med42‐8b (F1 = 0.768) and phi4 (F1 = 0.766). The lowest prevalence‐weighted average F1 scores were observed for gemma3 (F1 = 0.676) and hippomistral (F1 = 0.230).

**TABLE 3:** Prevalence‐weighted average F1 scores comparing large language models.

Model	Prevalence‐weighted average F1 score (95% CI)
llama3.3	0.845 (0.733, 0.927)
llama3‐med42‐70b	0.808 (0.618, 0.917)
OpenBioLLM‐Llama3‐70B	0.792 (0.698, 0.891)
mistral	0.772 (0.627, 0.860)
llama3‐med42‐8b	0.768 (0.639, 0.842)
phi4	0.766 (0.680, 0.851)
gemma3	0.676 (0.460, 0.782)
hippomistral	0.230 (0.121, 0.278)

*Note:* The prevalence‐weighted average F1 score is a summary measure of overall multi‐label performance that combines label‐specific F1 scores while weighting each label according to its prevalence in the dataset. This approach reflects aggregate performance across multiple outcomes while accounting for class imbalance, but should be interpreted alongside label‐specific metrics for clinically important outcomes. Values represent the mean prevalence‐weighted average F1 score with 95% confidence intervals derived from 1000 bootstrap samples. This summarises overall multi‐label performance across all classification tasks and should be interpreted as a comparative summary metric rather than as performance for any single outcome.

Abbreviation: CI, confidence interval.

Table 4 provides detailed diagnostic metrics for each classification category using the best‐performing model, llama3.3. The model showed high performance in identifying any injecting drug use (F1 = 0.897) and related substance use such as methamphetamine (F1 = 0.992), heroin (F1 = 0.973) and cannabis (F1 = 0.974). Performance was lower for categories such as prescription opioids (F1 = 0.400), benzodiazepines (F1 = 0.606) and historical injecting drug use (F1 = 0.505). Of note, some other models outperformed llama3.3 in specific domains (see Supplemental Table 5). For example, llama3‐med42‐70b and mistral showed higher F1 scores for classifying injecting drug use overall (0.951 and 0.947, respectively), although these differences were not statistically different given overlapping confidence intervals.

**TABLE 4:** Diagnostic metrics and 95% confidence intervals of best performing model (*llama 3.3*) *n* = 859.

Variable	F1 Score^b^	Accuracy	Sensitivity	Specificity	Positive predictive value	Negative predictive value
Injecting drug use	0.897 (0.855, 0.934)	0.967 (0.955, 0.979)	0.819 (0.753, 0.879)	0.999 (0.996, 1.000)	0.992 (0.972, 1.000)	0.963 (0.948, 0.977)
Current^a^	0.702 (0.639, 0.760)	0.909 (0.889, 0.928)	0.939 (0.889, 0.980)	0.905 (0.884, 0.926)	0.561 (0.486, 0.636)	0.991 (0.984, 0.997)
Historical^a^	0.505 (0.375, 0.614)	0.941 (0.923, 0.956)	0.929 (0.815, 1.000)	0.941 (0.924, 0.957)	0.347 (0.237, 0.459)	0.997 (0.994, 1.000)
Substances						
Heroin	0.973 (0.929, 1.000)	0.998 (0.994, 1.000)	0.973 (0.902, 1.000)	0.999 (0.996, 1.000)	0.973 (0.914, 1.000)	0.999 (0.995, 1.000)
Prescription opioids	0.400 (0.213, 0.556)	0.962 (0.949, 0.974)	0.786 (0.538, 1.000)	0.964 (0.953, 0.976)	0.268 (0.133, 0.415)	0.996 (0.991, 1.000)
Methamphetamine	0.992 (0.972, 1.000)	0.999 (0.997, 1.000)	0.984 (0.946, 1.000)	1.000 (1.000, 1.000)	1.000 (1.000, 1.000)	0.999 (0.996, 1.000)
Cannabis	0.974 (0.903, 1.000)	0.999 (0.997, 1.000)	1.000 (1.000, 1.000)	0.999 (0.996, 1.000)	0.950 (0.824, 1.000)	1.000 (1.000, 1.000)
Benzodiazepine	0.606 (0.456, 0.750)	0.970 (0.958, 0.981)	0.952 (0.850, 1.000)	0.970 (0.958, 0.981)	0.444 (0.300, 0.611)	0.999 (0.996, 1.000)
Opioid agonist therapy						
Methadone	0.962 (0.929, 0.988)	0.992 (0.985, 0.998)	1.000 (1.000, 1.000)	0.991 (0.983, 0.997)	0.926 (0.867, 0.977)	1.000 (1.000, 1.000)
Buprenorphine	0.833 (0.667, 0.947)	0.993 (0.986, 0.998)	1.000 (1.000, 1.000)	0.993 (0.986, 0.998)	0.714 (0.500, 0.900)	1.000 (1.000, 1.000)

*Note:* Values represent the mean and 95% confidence interval from 1000 bootstrap samples. Cocaine and fentanyl were evaluated as separate substance categories but are not reported due to low numbers (*n* = 3 for each).

^a^ Current or historical injecting drug use only.

^b^ F1 score is a summary measure of diagnostic performance that balances sensitivity and positive predictive value, reflecting a model's ability to correctly identify true cases while minimising false positives.

Supplemental Table 6 shows a description of the errors committed by Llama‐3. False negatives for identifying injecting drug use were most commonly due to missed documentation of historical (*n* = 17) or current (*n* = 10) injecting drug use, often due to subtle or indirect language in the clinical notes. For current injecting drug use, all 71 false positives resulted from cases lacking explicit mention of current use. Within historical injecting drug use, errors frequently arose from missed cue words indicating temporality (e.g., “previous” or “former”), confusion with hepatitis C‐related documentation, or lack of historical context. False positives in prescription opioids often occurred when opioid use was identified for pain management (*n* = 27) rather than misuse, with similar issues observed in *benzodiazepine* classification, where mentions of prescribed or historical use without illicit context led to false positives (*n* = 24).

Fairness analyses revealed age‐related differences in the best model's performance across multiple metrics (Table 5). Among participants aged 52 years or older, sensitivity was substantially lower (true positive rate, 0.536 [95% CI, 0.333–0.714]) than among those younger than 52 years (0.884 [95% CI, 0.824–0.938]), yielding a true positive rate ratio of 0.606, below the 0.80 fairness threshold. Predictive values and specificity were comparable by age (positive predictive value ratio, 1.000; negative predictive value ratio, 0.990; true negative rate, 1.000), although demographic parity was modestly lower in younger participants (demographic parity ratio, 0.784). Performance by sex was consistent across all fairness dimensions, with ratios ≥ 0.939 for all metrics. By Aboriginality, parity thresholds were met for sensitivity, specificity, predictive values and balanced performance. However, proportional parity was lower among Aboriginal and Torres Strait Islander participants (demographic parity, 0.579 [95% CI, 0.463–0.692]; demographic parity ratio, 0.656), indicating a lower overall predicted‐positive rate relative to the comparison group despite similar operating characteristics.

**TABLE 5:** Fairness analyses in identifying people who inject drugs.

Group				Actual metrics						Parity ratios					
	N	PWID	Non‐PWID	Demographic parity	TPR (Equality of opportunity)	TNR (Equalised odds)	PPV (Predictive parity)	NPV (Predictive parity)	MCC (Balanced performance)	DP ratio	TPR ratio	TNR ratio	PPV ratio	NPV ratio	MCC ratio
Age															
< 52 years	443	121	322	0.756 (0.715, 0.795)	0.884 (0.824, 0.938)	0.997 (0.990, 1.000)	0.991 (0.969, 1.000)	0.958 (0.935, 0.978)	0.914 (0.872, 0.952)	0.784 (0.739, 0.827)	1.000 (1.000, 1.000)	0.997 (0.990, 1.000)	0.991 (0.969, 1.000)	0.990 (0.961, 1.000)	1.000 (1.000, 1.000)
≥ 52 years	416	28	388	0.964 (0.944, 0.981)	0.536 (0.333, 0.714)	1.000 (1.000, 1.000)	1.000 (1.000, 1.000)	0.968 (0.949, 0.984)	0.720 (0.567, 0.839)	1.000 (1.000, 1.000)	**0.606 (0.378, 0.812)**	1.000 (1.000, 1.000)	1.000 (1.000, 1.000)	1.000 (0.982, 1.000)	**0.787 (0.619, 0.921)**
Sex															
Female	273	48	225	0.850 (0.805, 0.891)	0.854 (0.750, 0.939)	1.000 (1.000, 1.000)	1.000 (1.000, 1.000)	0.970 (0.948, 0.987)	0.910 (0.845, 0.962)	0.988 (0.926, 1.000)	1.000 (0.919, 1.000)	1.000 (1.000, 1.000)	1.000 (1.000, 1.000)	1.000 (0.984, 1.000)	1.000 (0.957, 1.000)
Male	586	101	485	0.860 (0.834, 0.885)	0.802 (0.726, 0.875)	0.998 (0.994, 1.000)	0.988 (0.958, 1.000)	0.960 (0.944, 0.976)	0.871 (0.818, 0.920)	1.000 (0.956, 1.000)	0.939 (0.809, 1.000)	0.998 (0.994, 1.000)	0.988 (0.958, 1.000)	0.990 (0.965, 1.000)	0.957 (0.878, 1.000)
Aboriginal and/or Torres Strait Islander status^a^															
Aboriginal and/or Torres Strait Islander	76	35	41	0.579 (0.463, 0.692)	0.886 (0.757, 0.977)	0.976 (0.919, 1.000)	0.969 (0.893, 1.000)	0.909 (0.814, 0.981)	0.870 (0.746, 0.971)	**0.656 (0.526, 0.787)**	1.000 (0.930, 1.000)	0.976 (0.919, 1.000)	0.969 (0.893, 1.000)	0.941 (0.840, 1.000)	0.990 (0.843, 1.000)
Non‐Indigenous	779	114	665	0.883 (0.859, 0.907)	0.798 (0.721, 0.870)	1.000 (1.000, 1.000)	1.000 (1.000, 1.000)	0.967 (0.952, 0.979)	0.878 (0.828, 0.923)	1.000 (1.000, 1.000)	0.901 (0.779, 1.000)	1.000 (1.000, 1.000)	1.000 (1.000, 1.000)	1.000 (0.980, 1.000)	1.000 (0.888, 1.000)

*Note:* Numbers denote point estimates with 95% bootstrap confidence intervals (1000 iterations). See Supplemental Table 4 for definitions and equations for these metrics. Ratios are calculated as the value for each level divided by the best‐performing level within the attribute. Values ≥ 0.80 are considered consistent with fairness under the 80% rule. Ratios in bold are below the 0.80 threshold.

Abbreviations: DP, demographic parity; MCC, Matthews correlation coefficient; NPV, negative predictive value; PPV, positive predictive value; PWID, people who inject drugs; TNR, true negative rate (specificity); TPR, true positive rate (sensitivity).

^a^ Four cases of unknown Aboriginality status were omitted from the analysis.

## 4 Discussion

This study evaluated the diagnostic performance of LLMs in identifying PWID and related clinical information, such as drug type and receipt of opioid agonist therapy, from EHR at a single hospital in Sydney, Australia. The best‐performing model (llama3.3) demonstrated high accuracy in identifying PWID overall, with particularly strong performance for current and common substances such as methamphetamine, heroin and cannabis. However, the model was less accurate in detecting historical injecting, benzodiazepines and prescription opioid use, highlighting important areas for improvement in classification performance.

This work expanded on previous work [10, 11] to identify meta‐data related to injecting drug use including the type of drug used and if opioid agonist therapy was provided, rather than focusing solely on identification of PWID. In addition, subgroup fairness analyses demonstrated generally consistent performance across sex and Aboriginal and Torres Strait Islander identification but highlighted reduced sensitivity among older patients, suggesting potential age‐related bias in the model's classification.

ICD‐based indicators of injecting drug use demonstrated high specificity but very low sensitivity, meaning that while individuals coded as PWID were almost certainly true cases, most patients with documented injecting drug use were missed. This finding highlights the inaccuracies of using ICD codes as research tools which has been documented previously in both PWID [8, 9, 10] and for other medical conditions, behaviours or sociodemographic circumstances [24, 25]. This is especially relevant when the substance use disorder is recorded as a secondary diagnosis during the hospitalisation, for example, on an infectious diseases ward admission where the primary diagnosis reflects the acute medical problem (e.g., endocarditis or pneumonia) and substance use is captured only as a comorbidity. In many research datasets that rely on administrative codes, only the primary diagnosis field is extracted, meaning that secondary diagnoses, including substance use disorders, may be systematically missed. Even when secondary codes are available, they are often inconsistently or incompletely recorded, especially for conditions perceived as sensitive or stigmatising [26]. Also of note, ICD codes for HIV were of no added value in identifying PWID, given the low prevalence of HIV among PWID in Australia (< 2%) [27] compared with Canada (11%) and the United States (8%) [28], where HIV codes have previously been tested [7, 11].

### 4.1 Clinical and Public Health Implications

These findings demonstrate the potential for NLP‐based approaches to augment or replace traditional coding systems for identifying PWID in hospital data, particularly for surveillance and research purposes. Accurate identification of PWID is critical for monitoring injection‐related infections, evaluating health system burden and supporting targeted public health interventions. The ability of LLMs to extract substance‐specific and treatment‐related information further enhances their potential utility for population‐level monitoring and service planning.

However, variability in performance across different types of information underscores that LLM outputs should be interpreted cautiously and validated against clinical context, particularly when extracting nuanced attributes such as temporality of use or illicit versus prescribed medication exposure.

### 4.2 Deployment Challenges

Effective deployment of NLP in a health system faces significant challenges. First, scalable computer infrastructure (e.g., dedicated on‐premise graphical processing unit [GPU] clusters) to support LLM inference and ongoing retraining is necessary [29]. Second, skilled personnel including data scientists and clinical informaticians are needed to develop, validate and maintain NLP pipelines [30]. Third, secure, governed access to unstructured clinical notes, requiring collaboration with information technology and medical records teams, is needed to build encrypted, de‐identified data feeds, implement role‐based permissions and ensure compliance with privacy regulations. In addition, deploying LLMs in real‐time clinical settings may be both technically challenging and expensive [31]. These constraints highlight the need for cost–benefit analyses and exploration of more efficient, task‐specific alternatives for routine use.

Injection drug use is a highly sensitive and stigmatised characteristic [26], and the application of LLMs to identify PWID raises important ethical considerations, particularly given the potential consequences of misclassification or unintended disclosure. Rigorous development and evaluation of such models are therefore essential to ensure that outputs are accurate, contextualised and used appropriately, especially when compared with existing approaches such as administrative coding that are known to be incomplete or inconsistently applied [8, 9, 10, 15]. Any implementation of NLP‐based surveillance tools must be accompanied by robust safeguards to minimise the risk of unintended misclassification or misuse [32]. These include strict data governance and access controls, use of de‐identified data wherever possible and clear limitations on secondary use of model outputs outside approved clinical, research, or public health purposes. Importantly, LLM‐derived classifications should not be used in isolation to make clinical or administrative decisions, but rather as decision‐support tools subject to human review and clinical judgement. Ongoing monitoring for bias, transparency in model development and evaluation, and engagement with community members are essential to ensure that such technologies are deployed in a manner that maximises benefit while minimising potential harm.

### 4.3 Limitations

This study had several limitations. First, this was a retrospective, single‐centre study conducted on discharge summaries from the infectious diseases service, which limit the generalisability of the findings to other specialties, institutions or geographic regions. Second, our gold‐standard annotations were performed by a single reviewer without inter‐rater reliability assessment, introducing potential bias, although each discordant classification was double checked. Third, off‐the‐shelf LLMs were applied without any task‐specific fine‐tuning which may have constrained their ability to capture idiosyncratic language use and contributed to errors such as misclassifying historical versus current use or failing to distinguish patient versus third‐party references. Supervised fine‐tuning or in‐context learning using labelled examples may improve performance and should be explored in future studies. Fourth, the relatively small number of cases for certain substances (e.g., fentanyl, cocaine) and treatment details limited our ability to robustly assess model accuracy across all drug categories. Fifth, overall model comparison relied in part on a prevalence‐weighted average F1 score as a summary metric for multi‐label performance. While useful for high‐level comparison across models, prevalence‐weighted aggregation can under‐emphasise performance on rare but clinically important outcomes. Finally, reliance solely on existing EHR documentation means that under‐documented or missing information could lead to false negatives, underscoring the need for prospective evaluation and integration with other data sources (e.g., laboratory results, pharmacy records) before clinical implementation.

### 4.4 Future Directions and Conclusion

Future work should focus on external validation across multiple hospitals and services, prospective implementation studies and exploration of hybrid approaches that combine NLP outputs with clinical workflows. Methodologically, exploration of supervised learning or in‐context learning approaches should be evaluated for improvement in diagnostic metrics especially for identifying the historical context of substance use, and substances that may have a licit use (e.g., prescription opioids).

In conclusion, LLMs can accurately identify PWID and extract key related information from unstructured hospital discharge summaries, outperforming traditional ICD‐code approaches in this setting. While performance was strong for the identification of PWID, our findings also highlight areas for improvement, particularly in extracting detailed substance and treatment information, which could be addressed through model fine‐tuning, enhanced prompts, integration with structured data, or stacking models. By embedding NLP‐driven surveillance tools into health systems, we could enhance research, guide targeted interventions and ultimately improve outcomes for this high‐risk population.

## Author Contributions

Each author certifies that their contribution to this work meets the standards of the International Committee of Medical Journal Editors.

## Funding

The authors have nothing to report.

## Conflicts of Interest

The authors declare no conflicts of interest.

## Supporting information


**Supplemental Table 1.** Annotator guide.
**Supplemental Table 2**. Model characteristics.
**Supplemental Table 3**. Prompt used to classify discharge summaries by large language models.
**Supplementary Table 4**. Fairness metrics used in the analysis.
**Supplemental Table 5**. F1‐score and 95% confidence intervals for selected models.
**Supplemental Table 6**. Error analysis.

## Data Availability

Unstructured text data is not available for sharing due to contractual obligations with St Vincent's Hospital. Code for all analyses are available at https://github.com/davigood1/nlp‐svh. This research was produced in whole or part by UNSW Sydney researchers and is subject to the UNSW Intellectual property policy. For the purposes of Open Access, the author has applied a Creative Commons Attribution CC BY licence to any Author Accepted Manuscript (AAM) version arising from this submission.
