# Supplementary material for: Large Language Models Accurately Identify People Who Inject Drugs From Infectious Diseases Discharge Summaries in an Australian Hospital
Source: Drug Alcohol Rev. 2026 May 14;45:e70169. doi: 10.1111/dar.70169 (PMC13175947; doi:10.1111/dar.70169)
Supplement: Supplementary file 1 — Supplemental Table 1. Annotator guide. Supplemental Table 2. Model characteristics. Supplemental Table 3. Prompt used to classify discharge summaries by large language models. Supplementary Table 4. Fairness metrics used in the analysis. Supplemental Table 5. F1‐score and 95% confidence intervals for selected models. Supplemental Table 6. Error analysis. [file DAR-45-0-s001.docx]

**Supplemental table 1. Annotator guide**

| **Variable** | **Values** | **Description** |
| --- | --- | --- |
| Injecting drug use (dichotomous) | Yes | There is a mention that the case injected drugs or has a history of injecting drug use. Terms may include: injection illicit drug use, injection ____ [specify drug], [specify drug] injector, IVDU, IVDA, PWID, IDU, intravenous drug user, intravenous drug abuser, injection drug user, skin popping, muscle popping. Key areas to look are in the History of Present Illness (HPI), social history, physical exam, and assessment and plan. |
|  | No | There is no mention that the case injected drugs. |
| Chronicity  (categorical) | Current: | Note reports that the case has injected an illicit substance within the last 30 days |
|  | Historical: | Note reports that the case has injected an illicit substance more than 30 days ago or has a history of using a injecting a drug without a current mention |
| Type  (categorical, multiple selection) | Heroin | Specified use of “heroin”. Other terms include “black tar”, “powder heroin”, “china white”, “dope”, “smack”, “horse”. |
|  | Prescription opioid | Specified illicit use of a prescription opioid. Examples include oxycodone, oxymorphone, hydrocodone, hydromorphone, Dilaudid, Opana, Norco, Percocet, Vicodin, oxys, methadone, buprenorphine. Fentanyl use should be classified with its own label. |
|  | Fentanyl | Specified injection, smoking or snorting of “fentanyl”. |
|  | Cocaine or crack | Specified injection, smoking, or snorting of “cocaine”, “crack” or “crack cocaine”. |
|  | Methamphetamine/amphetamine | Specified injection, smoking, or snorting of methamphetamine or amphetamines. Example terms include “meth”, “methamphetamine”, “amphetamine”, “speed”, “ice”, “crystal”. Notes may specify that the case used methamphetamine but not injected it. |
|  | Benzodiazepines | Specified illicit use of benzos, benzodiazepines, diazepam, valium, alprazolam, zanax, clonazepam, konipin |
|  | Cannabis/  marijuana | Specified use of cannabis, marijuana, THC, pot. |

**Supplemental table 2. Model characteristics**

| **Model Name** | **Domain** | **Family** | **Parameter size (billions)** | **Size (gigabytes)** | **Quantization** | **Average time per note (seconds)** |
| --- | --- | --- | --- | --- | --- | --- |
| phi4 | General | phi3 | 14.7 | 9.05 GB | Q4_K_M | 7.6 |
| mistral | General | llama | 7.2 | 4.11 GB | Q4_0 | 4.6 |
| gemma3 | General | gemma3 | 4.3 | 3.34 GB | Q4_K_M | 3.2 |
| llama3.3 | General | llama | 70 | 42.52 GB | Q4_K_M | 33.5 |
| thewindmom/  llama3-med42-70b | Medical | llama | 70 | 42.52 GB | Q4_K_M | 34.6 |
| thewindmom/  llama3-med42-8b | Medical | llama | 8 | 8.54 GB | Q8_0 | 4.8 |
| PrunaAI/  OpenBioLLM-Llama3-70B | Medical | llama | 70 | 49.95 GB | Q5_K_M | 36.3 |
| electricalgorithm/  hippomistral | Medical | Llama | 7 | 4.11 GB | Q4_0 | 4.1 |
| Note: All models listed above are in GGUF format and were run locally using the Ollama framework. | | | | | | |

**Supplemental table 3. Prompt used to classify discharge summaries by large language models**

| **Prompt development** | Large language models were evaluated using a zero-shot classification framework with a fixed, task-specific prompt. Prompt development was informed by concurrent work conducted in the United States on LLM-based substance use detection in clinical notes (Harel-Canada et al., 2025), which evaluated similar classification targets and exclusion criteria, and by the prespecified annotator guide used for manual gold-standard review in the present study.  The initial prompt structure and label definitions were adapted from this prior work and translated to align with the variables of interest in our study, including injection drug use, specific substances, temporality of use, and opioid agonist therapy. The prompt was then tailored to the Australian clinical context, incorporating locally relevant terminology, abbreviations, and documentation conventions (e.g., references to methamphetamine as “ice” or “CMA”).  Prompt development followed an iterative, expert-driven process. Approximately 3-4 candidate prompt variants were evaluated during pilot testing on a small subset of de-identified discharge summaries. Variants differed in the level of clinical specificity, explicit exclusion rules (e.g., prescribed versus illicit medication use), and handling of temporal language. Prompt variants were assessed qualitatively based on face validity, interpretability, and observed error patterns rather than quantitative optimization.  The final prompt was selected prior to model evaluation and applied unchanged across all models. No prompt tuning, prompt ensembling, or model-specific prompt customization was performed. |
| --- | --- |
| **Context** | You are a medical expert conducting important research where accuracy is essential. |
| **Task Description** | Determine whether the medical text contains reference to illicit drug use.  Specifically look for mentions of the following drugs:  Injection Drugs: Injection drug use (IDU, IVDA, IVDU) refers to the use of illicit drugs administered via needles, often associated with higher risks of infectious diseases.  General Drugs: General drug use refers to the use of any illegal or illicit substances.  Heroin: Heroin is an illegal opioid drug known for its high potential for addiction and overdose.  Cocaine: Cocaine is a powerful stimulant drug that is often abused for its euphoric effects.  Methamphetamine: Methamphetamine (including illicit amphetamine use, but not prescribed amphetamines for ADHD) is a potent central nervous system stimulant that is highly addictive. In Australia, doctors use the acronym CMA\|cma to denote methamphetamine use.  Benzodiazepine: Benzodiazepines (only if being misused or used illicitly, not if taken as prescribed) are a class of psychoactive drugs commonly prescribed for anxiety, insomnia, and other conditions but can be abused for their sedative effects. Some examples are diazepam (valium), clonazepam (klonipin), alprazolam (xanax). If a benzodiazepine is being prescribed for alcohol withdrawal or treatment of seizure this is not an example of illicit use and should be marked false.  Prescription Opioids: Prescription opioids (only if being misused or used illicitly, not if taken as prescribed) are medications typically prescribed for pain relief but can be highly addictive when misused. Don't confuse prescribed mentions of methadone or buprenorphine (e.g. suboxone) in this category. In Australia, a common one is misused prescription opioid is Endone and buprenorphine. If an opioid is prescribed for pain management this is not illicit use, and should be marked false.  Fentanyl: A high potency opioid responsible for many deaths.  Cannabis: Cannabis, also known as marijuana, is often used recreationally or medicinally but can be illegal depending on the jurisdiction. Look for abbreviations like THC.  Snorting: If there is a mention that the illicit drug is being used via snorting by nose or inhalation  Smoking: If there is mention that the illicit drug is being used via smoking  Current: This only qualifies injection drug use. Is there mention that the person has recently injected drugs. Words that denote current injection drug use include 'secondary', 'background', 'after', 'recently'.  Historical: This only qualifies injection drug use. Is there mention that the person has injected drugs but not currently. This is clearly in the past. Words that denote historical include 'history', 'ex'.  Buprenoprhine: Is there mention that the doctors prescribed buprenorphine (or suboxone/subutex/buvidal/sublocade) for treatment of opioid use disorder. If used illicitly this is false. Do not confuse with methadone.  Methadone: Is there mention that the doctors prescribed methadone for treatment of opioid use disorder. If used illicitly this is false. Do not confuse with buprenorphine/suboxone. |
| **Special notes** | 1. The mere mention of a drug is not sufficient. You are only looking for illicit use of the drug in the medical note. Do not assume that a drug is being used illicitly without some evidence.  2. If the text warns against the use of a particular drug, that does not mean the patient is actually using the drug.  3. If family drug use is present, that is not relevant to the patient and should not be flagged.  4. If the patient denies using a particular drug, do not mark that drug as being present. For example, if the note says 'patient denied using heroin', then the label should be False.  5. Many opioids and benzodiazepines are appropriately used and should not be noted. We only want you to identify cases where the patient is not using them appropriately. For example, if they are taking Percocets or Xanax acquired from friends or from the streets, this would be considered illicit misuse.  6. Medical recommendations about drugs do not mean the patient is actually using the drug. |

**Supplementary Table 4. Fairness metrics used in the analysis**

| Metric | Fairness principle | Operational definition | Formula |
| --- | --- | --- | --- |
| Demographic parity | Independence: predictions independent of group membership. | Equal predicted positive rates (proportion of individuals classified positive) across groups | **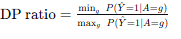** |
| Equality of opportunity | Separation: predictions independent of group membership conditional on true outcome. | Equal true positive rates (TPR) (sensitivity) across groups | **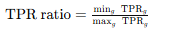** |
| Equalized odds | Separation: predictions independent of group membership conditional on true outcome. | Equal TPR and true negative rates (TNR, specificity) across groups | **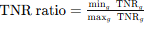** |
| Predictive parity | Sufficiency: outcome independent of group membership conditional on prediction. | Equal predictive values across groups | **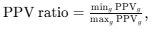**  **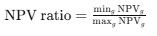** |
| Matthews Correlation Coefficient (MCC) | Balanced performance (all 4 confusion matrix cells) | Summary measure of classification quality for each group | **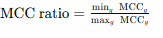** |

**Supplemental table 5. F1-score and 95% confidence intervals for selected models.**

| **Variable** | **Llama3.3** | **Llama3med42** | **Mistral** |
| --- | --- | --- | --- |
| **Injecting drug use** | 0.897 (0.855, 0.934) | 0.951 (0.925, 0.974) | 0.947 (0.917, 0.970) |
| Current | 0.702 (0.639, 0.760) | 0.591 (0.526, 0.650) | 0.716 (0.645, 0.788) |
| Historical | 0.505 (0.375, 0.614) | 0.378 (0.261, 0.476) | 0.454 (0.324, 0.571) |
| **Substances** |  |  |  |
| Heroin | 0.973 (0.929, 1.000) | 0.881 (0.795, 0.944) | 0.866 (0.772, 0.943) |
| Prescription Opioids | 0.400 (0.213, 0.556) | 0.286 (0.125, 0.431) | 0.256 (0.141, 0.374) |
| Fentanyl | 0.857 (0.000, 1.000) | 0.857 (0.400, 1.000) | 1.000 (1.000, 1.000) |
| Cocaine | 1.000 (1.000, 1.000) | 1.000 (1.000, 1.000) | 0.333 (0.000, 0.800) |
| Methamphetamine | 0.992 (0.972, 1.000) | 0.992 (0.971, 1.000) | 0.765 (0.666, 0.852) |
| Cannabis | 0.974 (0.903, 1.000) | 0.950 (0.870, 1.000) | 0.621 (0.375, 0.800) |
| Benzodiazepine | 0.606 (0.456, 0.750) | 0.618 (0.471, 0.742) | 0.538 (0.393, 0.667) |
| **Opioid agonist therapy** |  |  |  |
| Methadone | 0.962 (0.929, 0.988) | 0.961 (0.928, 0.988) | 0.831 (0.763, 0.889) |
| Buprenorphine | 0.833 (0.667, 0.947) | 0.291 (0.176, 0.407) | 0.475 (0.314, 0.613) |
| Numbers denote point estimates with 95% bootstrap confidence intervals (1,000 iterations). | | | |

**Supplemental table 6. Error analysis**

| **Error category** | **Categorized Reason** | **False Positives** | **False Negatives** |
| --- | --- | --- | --- |
| **Injecting drug use** | | | |
| Missed documentation | Missed history of injecting drug use | 0 | 17 |
| Missed documentation | Missed current injecting drug use | 0 | 10 |
| Human annotation error | Human error | 1 | 0 |
| **Current Injecting drug use** | | | |
| Temporal misclassification | No explicit mention of current | 71 | 0 |
| Missed documentation | Missed background of IVDU | 0 | 4 |
| Human annotation error | Human error | 1 | 0 |
| Temporal misclassification | Missed 6 months without current use | 0 | 1 |
| Temporal misclassification | Missed relapse | 0 | 1 |
| **Historical Injecting drug use** | | | |
| Temporal misclassification | Missed word suggesting current | 24 | 0 |
| Missed documentation | No mention of injecting drug use | 9 | 0 |
| Contextual misinterpretation | May have confused HCV | 6 | 0 |
| Missed documentation | No mention of historical | 6 | 0 |
| Human annotation error | Human error | 2 | 0 |
| Temporal misclassification | Missed mention of history or in the past | 0 | 2 |
| Temporal misclassification | No explicit mention of historical | 2 | 0 |
| **Heroin** | | | |
| Missed documentation | Missed heroin | 0 | 1 |
| Human annotation error | Human error | 1 | 0 |
| **Prescription opioids** | | | |
| Contextual misinterpretation | Identified opioids, but was for pain | 27 | 0 |
| Contextual misinterpretation | Identified discussion about opioids | 1 | 0 |
| Contextual misinterpretation | Identified the prescription of naloxone for overdose prevention | 1 | 0 |
| Contextual misinterpretation | Misidentified pregabalin abuse as an opioid | 1 | 0 |
| Missed documentation | Missed injecting buprenorphine | 0 | 1 |
| Missed documentation | No mention of prescription opioids | 2 | 0 |
| **Fentanyl** | | | |
| Contextual misinterpretation | Fentanyl for pain, not illicit | 1 | 0 |
| **Methamphetamine** | | | |
| Missed documentation | Missed patient was referred to stimulant treatment services | 0 | 1 |
| **Benzodiazepines** | | | |
| Contextual misinterpretation | Identified benzodiazepine, but not illicit | 24 | 0 |
|  | Missed illicit diazepam use | 0 | 1 |
| Contextual misinterpretation | Identified urine benzodiazepines but no mention of illicit use | 1 | 0 |
| Temporal misclassification | Previous benzodiazepine use | 2 | 0 |
| Human annotation error | Human error | 1 | 0 |
| **Buprenorphine** | | | |
| Contextual misinterpretation | Identified suggestion for future buprenorphine use | 1 | 0 |
| Contextual misinterpretation | Illicit buprenorphine use | 1 | 0 |
| Temporal misclassification | Previous Buvidal use | 1 | 0 |
| Human annotation error | Human error | 1 | 0 |
| **Methadone** | | | |
| Contextual misinterpretation | Patient declined methadone | 5 | 0 |
| Temporal misclassification | Previous methadone use | 2 | 0 |
